# Drug monomers from *Salvia miltiorrhiza Bge*. promoting tight junction protein expression for therapeutic effects on lung cancer

**DOI:** 10.1038/s41598-023-50163-8

**Published:** 2023-12-21

**Authors:** Guanghui Zhu, Daorui Li, Xueqian Wang, Qiujun Guo, Yuanchen Zhao, Wei Hou, Jie Li, Qi Zheng

**Affiliations:** Oncology Department, Guang’anmen Hospital, China Academy of Chinese Medical Sciences, Beijing, 100053 China

**Keywords:** Cancer, Drug discovery

## Abstract

*Salvia miltiorrhiza Bge.* is a traditional Chinese medicine (TCM) that has been used for treatment of various diseases, including cancer by activating blood circulation and removing blood stasis. Tanshinone (TanIIA) and cryptotanshinone (CPT) are major lipophilic compounds extracted from the root of *Salvia miltiorrhiza Bge*., which are considered to be the effective compounds affecting the efficacy of the anti-tumor therapy of *Salvia miltiorrhiza Bge*. We have explored the mechanism of CPT and TanIIA exerting inhibition in non-small cell lung cancer (NSCLC) to provide experimental data support for guiding the translational development and clinical application of anti-tumor components of TCM. The subcutaneous tumor model and in vitro culture model of A549 cells was constructed to evaluate CPT and TanIIA's tumour-inhibitory effect respectively. RNA sequencing (RNA-seq) and bioinformatics analysis were conducted to identify differentially expressed genes (DEGs) and signalling pathways related to CPT and TanIIA treatment. qRT-PCR and Western blot were used to explore the mechanism of CPT and TanIIA intervention on NSCLC. Both CPT and TanIIA significantly inhibited the proliferation of A549 tumor cells and tumor growth in animal models. After intervention, the migration ability decreased and the level of apoptosis increased. RNA-seq results showed that both CPT and TanIIA could cause gene differential expression, miR-21-5p as one of the most significant gene expression differences between the two groups, and could act on cell connectivity. CPT and TanIIA play a regulatory role in regulating tight junction proteins (Occludin and ZO1), and Occludin mRNA and protein levels were reduced in an in vitro miR-21-5p overexpression A549 cell model. The mechanisms may be related to the reduction of miR-21-5p expression to increase the level of promoted tight junction protein expression for the purpose of inhibiting proliferation and invasion of NSCLC.

## Introduction

Lung cancer is the leading cause of cancer death among male and female worldwide^1^. It has been proved that traditional treatment and surgery are difficult to control lung cancer, and the mortality rate is 80–85% within 5 years. Although we have made great progress in understanding the molecular mechanism of lung cancer, the therapeutic intervention of lung cancer still needs further optimization^2,3^. Traditional chemotherapy also has the disadvantage of cytotoxicity to normal tissues. Therefore, finding effective and safe drugs to prevent, suppress or reverse the occurrence of lung cancer is still the focus of lung cancer research.

MicroRNAs (miRNAs/miRs) are small non-coding RNA molecules with a length of about 22 nucleotides, which come into the limelight as they play a role in RNA silencing and post-transcriptional gene regulation^4^. miRNAs are essentially in the process of tumorigenesis by regulating cell proliferation, metastasis, apoptosis, cell cycle, angiogenesis and metabolism. Most important of all, it regulate gene expression via post-transcriptional regulation of mRNA^5^. The main miRNAs involved in lung cancer are miR-21-5p, miR-92a-3p, miR-30b-5p, miR-191-5p, miR-484, miR-328-3p, miR-30c-5p, etc^6^.

Traditional Chinese medicine (TCM) are rich in active ingredients, which can be used for the treatment of human diseases and drug research and development. *Salvia miltiorrhiza Bge*., named as “Danshen” in Chinese, is the roots and rhizomes of *Salvia miltiorrhiza* Bge. (Labiatae)^7^ It contains a variety of chemical components, among which tanshinones are a kinds of phenanthraquinone derivative, CPT and TanIIA such which has attracted much attention in recent years because of its potential role in treating cancers^8–11^. Previous studies have demonstrated that CPT derived from *Salvia miltiorrhiza Bge*. can induce apoptosis and enhance the sensitivity of ovarian cancer cells to chemotherapy, but its effects related to the inhibition of lung cancer are less studied^12^. Phenoquinone structures bind to DNA molecules, and furan structures produce free radicals, which cause DNA damage in tumor cells and inhibit DNA synthesis. Most of these compounds have ternary or tetracyclic o-quinone or p-quinone structures on the skeleton (Wei et al.^13^), giving them antitumor, anti-inflammatory and antimicrobial activities (Bai et al.^14^; Chen et al.^15^; Ruan et al.^16^; Teng et al.^17^). Danshen has been shown to have a wide range of pharmacological activities, such as anti-tumor and anti-inflammatory^16,18,19^. The technology of extraction, synthesis and modification of Danshen has been mature. Therefore, Danshen, as a new anti-tumor drug, can reduce the economic cost of society and people.

**Figure 1:**
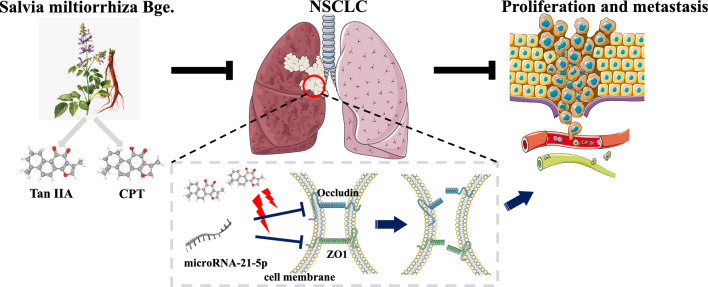
Workflow for the the potential mechanism of action of CPT and TanIIA on the NSCLC.

In this study, we focused on tanshinones compounds, and targeted two main compounds, CPT and TanIIA, which were detected to have the effects of inhibiting the proliferation, migration, apoptosis and cell cycle of A549 cells. Next, miRNA was analyzed by high-throughput sequencing, analyses to their counterpart proteins. Furthermore, Gene ontology (GO) and kyoto encyclopedia of genes and genomes (KEGG) pathway analyses were investigated using the identified target genes^20,21^. Results found that CPT and TanIIA had regulatory effect on miR-21-5p. As the biomarker and prognostic indicator of lung cancer, transient enforced expression of miR-21-5p mimics was transfected in A549 cells. It was found that miR-21-5p contributes differentiation of A549 cells through inhibiting the expression of Occludin. In addition, it was suggested that CPT and TanIIA might inhibit miR-21-5p to promote occludin protein expression, thereby refining the tight junction structure of tumour vascular endothelial cells and inhibiting metastasis of lung cancer cells. The potential mechanism of action of CPT and TanIIA on the lung cancer was presented in Fig. 1.

## Methods

### Drugs and antibodies

Cryptotanshinone (lot: 110852; > 99% purity) and tanshinone IIA (lot: 110766; > 98.9% purity) were purchased from China National Institute for Food and Drug Control. RPMI-1640 medium, foetal bovine serum (FBS), and trypsin were purchased from Gibco BRL (Grand Island, NY, USA). Antibodies ZO-1 (D7D12) rabbit mAb (8193) and GAPDH (D16H11) XP^®^ Rabbit mAb (5174), anti-rabbit IgG horseradish peroxidase (HRP)-linked antibody (7074) was purchased from Cell Signaling Technology (Danvers, MA, USA). Anti-Occludin antibody [EPR20992] (ab216327), anti-VEGFA antibody (ab46154), and anti-Ki67 antibody [37C7-12] (ab245113), goat anti-mouse IgG H&L (Alexa Fluor^®^ 647) (ab150115), and goat anti-rabbit IgG H&L (Alexa Fluor^®^ 488) (ab150077) were purchased from Abcam (Cambridge, MA, USA). qRT-PCR kits and reverse transcription kits were purchased from TOYOBO Co., Ltd. (Osaka, Japan). PCR primers were synthesized by Wuhan Servicebio Technology Co., Ltd (Wuhan, China). WB kits were purchased from Applygen Technologies Inc. (Beijing, China).

### Animal experiments and tissue harvest

All animal experiments were previously approved by an animal care committee (The ethics committee of the Guang'anmen Hospital, China Academy of Chinese Medical Sciences, No. 2020-011-SQ) and performed in accordance to the national guidelines for animal experiments. All animal experiments were performed in accordance to the ARRIVE guidelines. 6–8 weeks male Balb/c-nu/nu nude mice were purchased from Beijing Vital River Laboratory Animal Technology (Beijing, China) and housed in Peking university health science center department of laboratory animal science (SPF level, 22 ± 2 °C, 55 ± 5% relative humidity with a 12 h light/dark cycle). The mice were free to food and water. 1 × 10^6^ A549 cells were inoculated subcutaneously into the mice^22^. The 36 model mice were randomly divided into control group (n = 6, normal saline, once a day), bevacizumab group (n = 6, 5 mg/kg/day), high-dose CPT group (n = 6, 10 mg/kg/day), low-dose CPT group (n = 6, 5 mg/kg/day), high-dose TanIIA group (n = 6, 10 mg/kg/day), low-dose TanIIA group (n = 6, 5 mg/kg/day). The inoculation site of mice was observed daily. The dosage of CPT and TanIIA in animals is referred to the preliminary experiments and literature reports^23–25^. The longest diameter (a) and shortest diameter (b) of the tumor were measured with vernier caliper, and the tumor volume was calculated as V (mm^3^) = 1/6π ab^2^. The pathological changes of transplanted tumor in mice were observed. On the 21st day after modeling, transplanted mouse tumors were taken, fixed with paraformaldehyde, dehydrated, embedded in paraffin, and sliced. The changes of inflammatory cell infiltration and tumor cell morphology were observed by HE staining light microscope.

### Cell culture and management

A549 cells were cultured in RPMI-1640 medium supplemented with 10% FBS, 100 U/ml penicillin, and 100 mg/ml streptomycin at 37 °C in a 5% CO_2_ atmosphere, were obtained from the National Infrastructure of Cell Line Resource (Beijing, China) and cultured. CPT was dissolved in DMSO to prepare 10 mg/ml (34 mM) and TanIIA was dissolved in DMSO to prepare 5 mg/ml (17 mM) stock solutions for the in vitro experiments. The cell viability was measured with the CCK-8 assay. A concentration of 6 μg/ml CPT (20.2 μM) and 4 μg/ml (13.6 μM) TanIIA was added to the cell for subsequent experiments.

### Cell viability assay

The proliferation and cytotoxicity of cells were determined using a CCK-8 assay (Dojindo Laboratories, Kumamoto, Japan). A total density of approximately 4000 cells/well A549 cells were seeded in 96-well plates for 24 h. The cells were treated with different concentrations of CPT and TanIIA in 100 μL per well and incubated at 37 °C, 5% CO_2_ for 24 and 48 h. Subsequently, 10 μl of CCK-8 reagent was added to each well and incubated at 37 °C and 5% CO_2_ for 2 h. The absorbance was measured at a wavelength of 450 nm.

### Apoptosis assay

A549 cells were plated in 6-well plates at a density of 5 × 10^5^ cells/well and incubated with 0, 6 μg/ml CPT and 4 μg/ml TanIIA for 24 h at 37 °C. The relative amount of Annexin V-fluorescein isothiocyanate-positive/propidium iodide-negative cells were detected using an AnnexinV-FITC/PI Kit I (556547, BD Biosciences, Franklin Lakes, NJ, USA) and detected and analyzed using Flow cytometry (BD Biosciences, Franklin Lakes, NJ, USA).

### Wound-healing assay

A549 cells, in 2 mL of RPMI medium containing 10% FBS, were cultured in 6-well cell culture plates for 24 h. After the cells formed a fused monolayer, they were scored with a pipette tip and cultured in medium containing CPT (6 μg/ml) and TanIIA (4 μg/ml) for 24 h and 48 h. Then, the distance of the scratch was examined at the beginning of the experiment and 24 h and 48 h later.

### IF staining and analysis

The cells were seeded in 24-well plates at a density of 5 × 10^5^ cells per well in 500 μl of medium. After the cells were treated with different methods, cells at 80% confluence on cover slips were fixed with 4% paraformaldehyde. The samples were incubated with Occludin (1:200, ab216327, abcam, Cambridge, MA, USA) and ZO1 (1:200, 8193, Cell Signaling Technology, Danvers, MA, USA) at 4 °C for overnight. Subsequently, the samples were washed 3 times with PBST and incubated with the secondary antibody (ab150115, ab150077, abcam, Cambridge, MA, UK) at 37 °C for 60 min. The samples were counter-stained with DAPI and maintained at 4 °C. Measurements were made at 400× magnification using an ocular grid.

### mRNA profile detection

Total RNA extraction was done from A549 after different treatments with TRIzol reagent. The mRNA profile of cells in different treatment groups was analyzed by RNA sequencing (RNA-Seq), Differentially expressed genes were determined based on Q value (Adjusted P-value) ≤ 0.05^26^. Isolated RNA samples were sent to BGI Co., LTD. in Shenzhen for mRNA-seq using BGISEQ-500.

### Analysis of functional and pathway enrichment

Biological function Gene Ontology (GO) and Kyoto Gene and Genome Encyclopedia (KEGG) signal pathway enrichment analysis were performed using the online platform “Dr. Tom 2.0” designed by BGI Tech Co., LTD. Among them, GO enrichment analysis results come from the following three databases: Uniprot, gene2GO and idmapping. KEGG pathway enrichment analysis was retrieved from KEGG 93.0.

### Cell transfection

The cells were distributed into a six-well plate according to the density of 5 × 10^5^ cells/ml per well. In about 24 h, miR-21-5p mimics vectors were used and transfected into cells using a lipo3000 kit (Invitrogen, Shanghai, China), the dosages of lipo3000: miR-21-5p mimics = 3(μl): 3(μg). After gene transfection, the cells were subsequently cultured for 48 h and harvested for qRT-PCR and WB detection.The primers of miR-21-5p mimics oligoribonucleotides were listed in Table 1. The reference gene sequences were cleaned and sorted by Bowtie2, and then the gene expression level data were calculated by RSEM. Finally, the differentially expressed genes (DEGs) were obtained by DESeq2 analysis (Q value ≤ 0.05) (Fig. 5A). GO and KEGG pathway analysis was used to understand the biological processes and pathways that enriched A549 cells after CPT or TanIIA treatment. Detailed results are shown in Tables S4–S7. Based on Q values, we demonstrate the top 20 terms from small to large.

**Table 1:** The sequences of the RNA oligoribonucleotide.

Name		Sequences (5′–3′)
hsa-miR-21-5p mimics	Sense	UAGCUUAUCAGACUGAUGUUGA
	Anti‐sense	UCAACAUCAGUCUGAUAAGCUA
mimics NC-21 bp	Sense	UUGUACUACACAAAAGUACUG
	Anti‐sense	GUACUUUUGUGUAGUACAAUU

#### Western blot analysis

Proteins are extracted and quantified according to procedures specified by the manufacturer of the reagent used. Protein extractant (50 mmol/L Tris–HCL, pH 7.4; 10% SDS; 1 mmol/L PMSF, 0.25% sodium deoxycholate) were extracted and placed on ice for 30 min. Then, the supernatant was collected by centrifugation at 12,000 rpm for 10 min at 4 °C and the protein concentration was determined by BCA method. The 20 μg protein samples were separated by 5% and 10% SDS–polyacrylamide gel electrophoresis (PAGE) and then transferred to polyvinylidene fluoride (Millipore, USA) membrane by electrotransfer. Using rabbit ZO1 (1:10,000, 8193, CST, Danvers, MA, USA), rabbit occludin (1:3000, ab216327, Abcam,Cambridge, MA, USA), rabbit anti-VEGFA (1:10,000, ab46154, Abcam,Cambridge, MA, USA), rabbit anti-GAPDH polyclonal antibody (1:5000, 5174, CST, Danvers, MA, USA). The blots were cut prior to hybridisation with different antibodies. PVDF membrane was incubated in a 4 ℃ closed solution. Finally, these membranes were incubated with an anti-rabbit IgG-HRP linked antibody (1:5000,7074, abcam, Cambridge, MA, UK) at room temperature for no less than 60 min and with an electrochemiluminescence (ECL) reagent for about 30 s. The treated PVDF membranes were exposed to X-rays and photographed with the BIO-RAD ChemiDoc XRS gel imaging system. Export the image and analyze it using ImageProPlus 4.5 software (Media Cybernetics, Bethesda, MD, USA).

### qRT-PCR analysis

Total RNA was isolated from approximately 1 × 10^6^ of A549 cells according to the manufacturer’s instructions. RNA samples with an OD260/OD280 ratio of 1.9–2.1 and an OD260/OD230 ratio greater than 2.0 were used for the analysis. The cDNA was synthesized from total RNA by the reverse transcription of 1 μg of total RNA using the reverse transcription kit (FSQ-101, TOYOBO Co., Ltd., Osaka, Japan). The Premier 5.0 software was used to design PCR primer sequences based on GenBank sequences, which were shown in Table 2. Polymerase chain reaction was performed at 95 °C for 15 min, followed by denaturation at 95 °C for 10 s, annealing at 60 °C for 30 s, extension at 72 °C for 31 s (Foster Applied Biological Systems 7300 USA), 40 cycles. The relative quantitative analysis was performed by ^2−ΔΔ^Cq method^27^.

**Table 2:** Primer sequences list of qRT-PCR.

Primer name		Sequences (5′–3′)
H-ZO-1	Sense	TTCCAGCCAGCCTGCTAAAC
	Anti‐sense	CAATAGCGTAGCCCGTTCATCT
H-Occludin	Sense	TTCCTATAAATCCACGCCGG
	Anti‐sense	TGTCTCAAAGTTACCACCGCTG
H-GAPDH	Sense	GGAAGCTTGTCATCAATGGAAATC
	Anti‐sense	TGATGACCCTTTTGGCTCCC
hsa‐U6	Sense	CTCGCTTCGGCAGCACA
	Anti‐sense	AACGCTTCACGAATTTGCGT
hsa‐miR‐21‐5p	Stem loop	CTCAACTGGTGTCGTGGAGTCGGCAATTCAGTTGAG TCAACATC
	Sense	ACACTCCAGCTGGGTAGCTTATCAGACTGA
	Anti‐sense	TGGTGTCGTGGAGTCG

### Statistical analyses

Statistical analysis and mapping involved in this study were performed using GraphPad Prism8 (GraphPad Software, San Diego, CA, USA). When the normal distribution and variance were equal for each group, the data were expressed as mean standard deviation (SD) and calculated using SPSS version 25.0 (SPSS Inc., Chicago, IL, USA). When the data involved multi-group comparisons and post-hoc comparisons, one-way Analysis of variance (ANOVA) test and Tukey's multiple comparison test were used, respectively. We stipulate that when two-sided *P*-value < 0.05, the difference was considered statistically significant.

## Results

### Effects of CPT and TanIIA on tumor proliferation of A549 transplanted tumor mice

**Figure 2:**
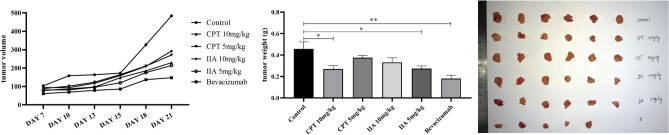
Identifying the intervention effect of CPT and TanIIa on A549 cell lung cancer. The inoculation site of mice was observed daily. The longest diameter (a) and shortest diameter (b) of the tumor were measured with vernier caliper, and the tumor volume was calculated as V (mm3) = 1/6π ab^2^. On the 21st day after modeling, transplanted mouse tumors were taken and weighed. The tumor volume growth of 10 mg/kg or 5 mg/kg CPT, and TanIIA 10 mg/kg or 5 mg/kg groups were lower than that of the control group. On the 21st day after inoculation, the tumor weight of CPT 10 mg/kg or 5 mg/kg, and TanIIA 10 mg/kg or 5 mg/kg groups were lighter than that of the control group.

The tumor volume growth of 10 mg/kg or 5 mg/kg CPT, and TanIIA 10 mg/kg or 5 mg/kg groups were lower than that of the control group. On the 21st day after inoculation, the tumor weight of CPT 10 mg/kg or 5 mg/kg, and TanIIA 10 mg/kg or 5 mg/kg groups were lighter than that of the control group (Fig. 2).

**Figure 3:**
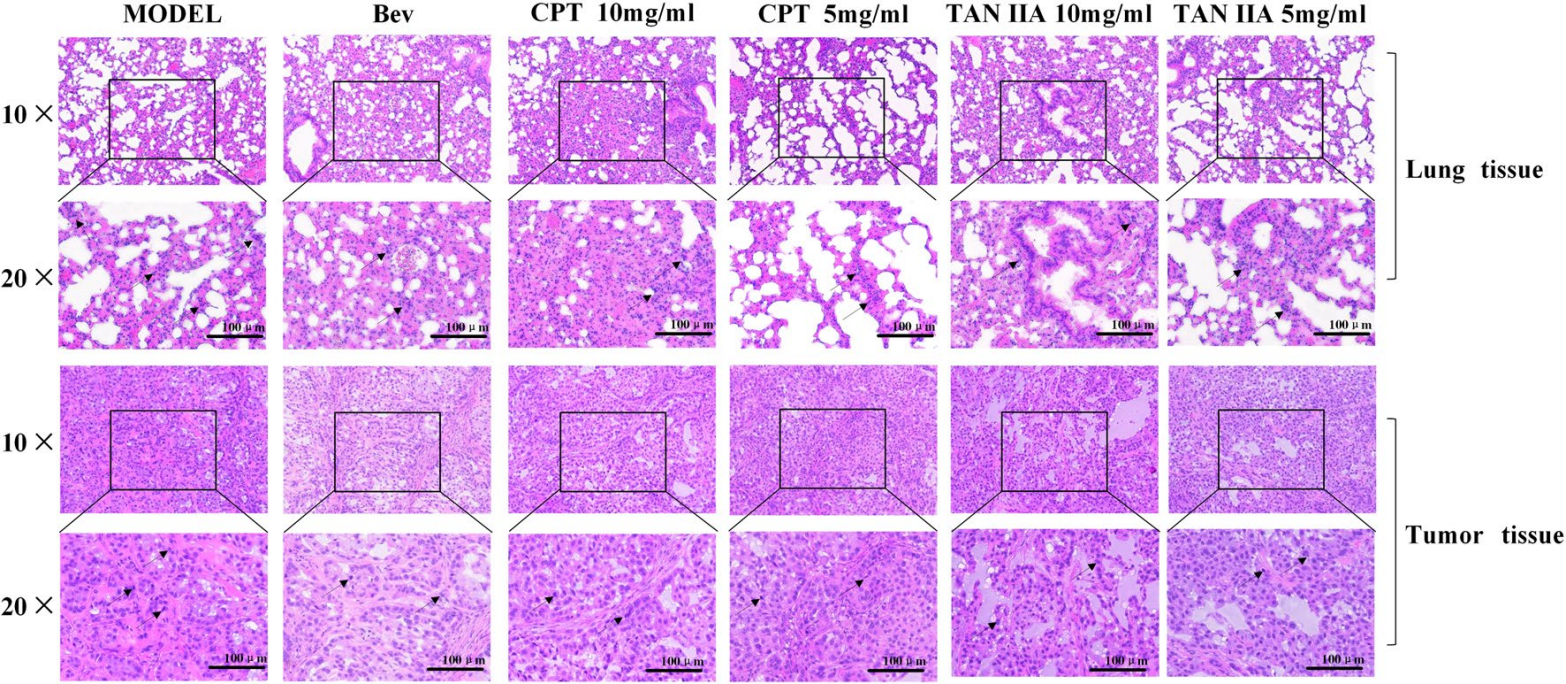
Comparison of HE of lung and tumor tissue in various groups of mice. The pathological changes of transplanted tumor in mice were observed. On the 21st day after modeling, transplanted mouse tumors were taken, fixed with paraformaldehyde, dehydrated, embedded in paraffin, and sliced. The changes of inflammatory cell infiltration and tumor cell morphology were observed by HE staining light microscope. The results of HE stained sections of lung and tumor tissue of the mice showed that the control group formed micro metastases accompanied by a large number of inflammatory cell infiltration. However, the incidence of metastasis was low under the intervention of drugs, a small number of mouse lungs also developed microscopic metastases. It could be seen that the tumor cells grow strongly in the tumor tissue of the control group, the cells are dense or formed into large sheets. And, the nuclei divided more, part of the tumor cells infiltrated into the nearby fat layer and muscle tissue. The blood vessels were abundant, and the necrotic area was small. In the administration group, the above indexes were mild and accompanied by a large number of lymphocyte and macrophage infiltrates.

The results of HE stained sections of lung and tumor tissue of the mice showed that the control group formed micro metastases accompanied by a large number of inflammatory cell infiltration. However, the incidence of metastasis was low under the intervention of drugs, a small number of mouse lungs also developed microscopic metastases. It could be seen that the tumor cells grow strongly in the tumor tissue of the control group, the cells are dense or formed into large sheets. And, the nuclei divided more, part of the tumor cells infiltrated into the nearby fat layer and muscle tissue. The blood vessels were abundant, and the necrotic area was small. In the administration group, the above indexes were mild and accompanied by a large number of lymphocyte and macrophage infiltrates (Fig. 3).

### CPT and TanIIA inhibits the proliferation, migration and apoptosis of A549 cells

**Figure 4:**
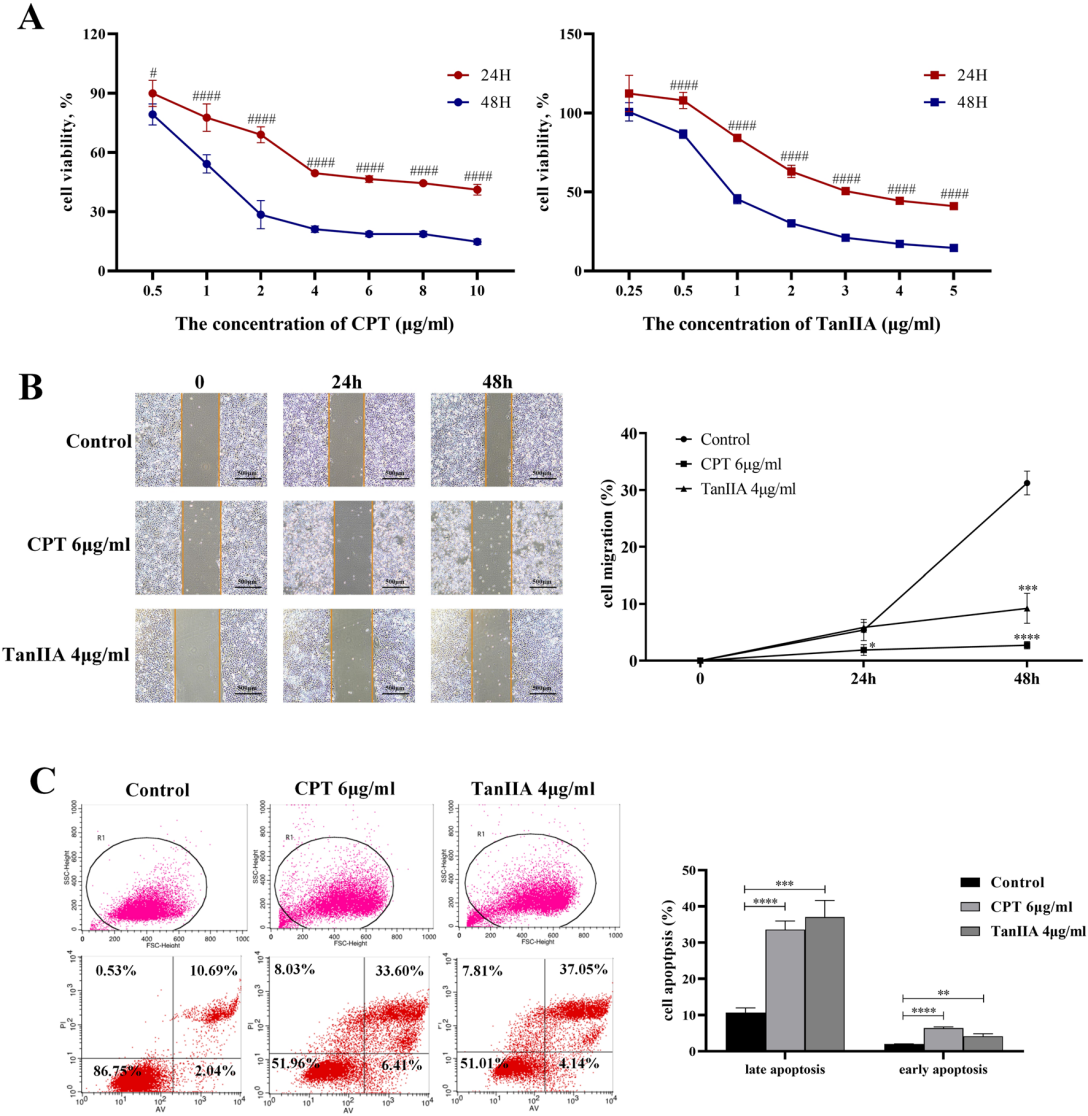
Inhibitory effect of CPT and TanIIA on A549 cell viability, migration, apoptosis (**A**) A549 cells were treated by CPT and TanIIA of indicated concentrations for 24 h, and 48 h. (**B**) Wound healing assay in A549 cells grown in monocultures or co-cultures treated with CPT and TanIIA. It represented invert phase-contrast microscope images showing the scratch (wound) at time 0 h, 24 h, and 48 h with/without exposure to CPT or TanIIA. Magnification ×40. Quantitative analysis of the percentage of wound healing in A549 cells after treatment with CPT or TanIIA. The values were normalized by the wound width at the same area of the scratch at time 0 h. (**C**) Early apoptotic cells are Annexin V-positive and PI-negative (Annexin V-FITC+/PI−), whereas late (end-stage) apoptotic cells are Annexin V/PI-double-positive (Annexin V-FITC+/PI+) Treatment with 6 μg/ml CPT and 4 μg/ml TanIIA for 24 h significantly increased late apoptosis and early apoptosis of A549 cells. ####*P* < 0.0001, #*P* < 0.05, compared with 48 h were recognized as statistically significant. ****P < 0.0001, ***P < 0.001, **P < 0.01, *P < 0.05, compared with control group were recognized as statistically significant.

Significantly, as shown in Fig. 4A, CPT and TanIIA significantly diminished the proliferative activities of A549 cells in a time- and dose-dependent manner compared with the control group. The IC50 values (the concentration of drug inhibiting 50% of the cells) of CPT in A549 cells at 24 h, and 48 h were around 6 μg/ml and 1.4 μg/ml (20.2 μM and 4.7 μM), respectively. While the IC50 values of TanIIA in A549 cells at 24 h, and 48 h were around 3.7 and 1.5 μg/ml (12.6 μM and 5.1 μM), respectively. According to the viability curve, we choose 6 μg/ml (20.2 μM) of CPT and 4 μg/ml (13.6 μM) of TanIIA administrated for 24 h as the optimum concentration range in the following experiments.

Next, we assessed treated with CPT and TanIIA may have any influence on migration using a wound healing assay. The values were normalized by the wound width at the same area of the scratch at time 0 h. The percentage of cell migrated of CPT and TanIIA treatment was 2.72% and 9.21%, which was markedly decreased percentage of cell migrated, compared with the control group 31.23% for 48 h shown in Fig. 4B.

To investigate the role of CPT and TanIIA upregulation on apoptosis in A549 cells, the apoptosis rate was tested by Annexin V-FITC/PI double staining. We found that CPT and TanIIA remarkable increased the late apoptosis and early apoptosis of A549 cells in control groups (Fig. 4C).

### Changes in gene expression under different treatments analyzed by mRNA-Seq

A total of 12 samples were sequenced on DNBSEQ platform, with an average yield of 32.84 Mb reads per sample. The average alignment ratio of the sample comparison genome was 92.55%. A total of 2019 miRNAs were detected.

#### Identification of DEGs and functional enrichment

**Figure 5:**
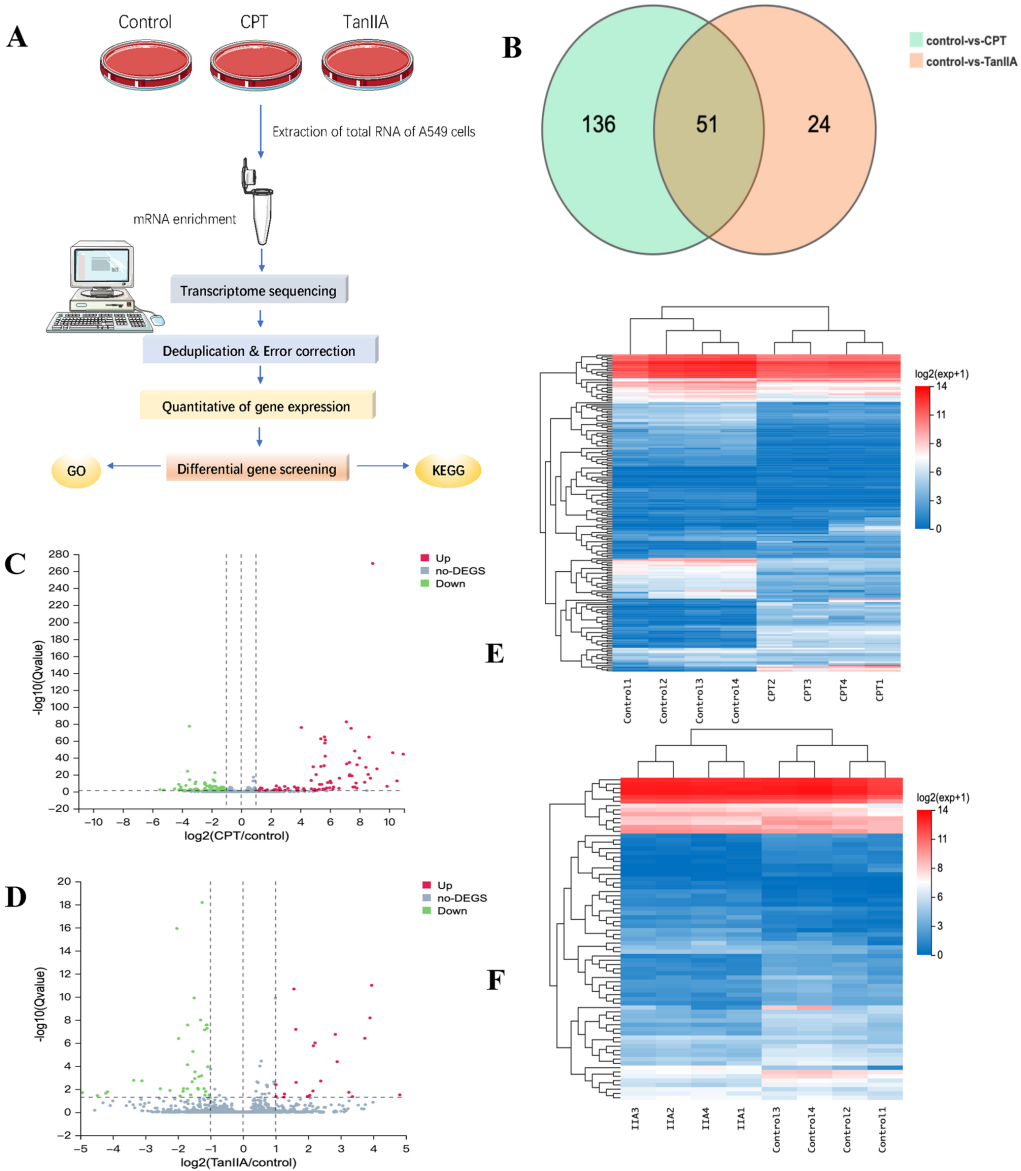
mRNA-seq analysis of changes in gene expression under different treatments (**A**) Schematic diagram of the mRNA transcriptome sequencing process. (**B**) Venn interactions between control and CPT groups and control and TanIIA group DEGs. (**C**) Volcano plot of control versus CPT group DEGs. (**D**) Volcano plots of control versus TanIIA group DEGs. (**E**) Heat map view of DEGs in the control group versus the CPT group. (**F**) Heat map view of DEGs in the control group versus the TanIIA group.

There were 187 differentially expressed genes between the control and CPT groups, and 75 genes were differentially expressed between the control and TanIIA groups. In addition, we plotted Venn diagrams showing that CPT and TanIIA could act on 51 DEGs simultaneously (Fig. 5B, Tables S1–S3).

When A549 cells were tested to compare changes in gene expression before and after CPT intervention, a total of 109 DEGs were found to be up-regulated, while 78 DEGs were down-regulated (Fig. 5C). We also compared the changes in gene expression in A549 cells before and after the TanIIA intervention and found that 35 genes were up-regulated and 40 DEGs were down-regulated (Fig. 5D). After obtaining the up- and down-regulated genes in the two groups before and after the intervention respectively, we drew clustering heatmaps based on the z-scores column orientation shown in Fig. 5E and F respectively. miR-21-5p was found to be ranked as one of the differentially expressed genes in both the CPT and TanIIA groups.

**Figure 6:**
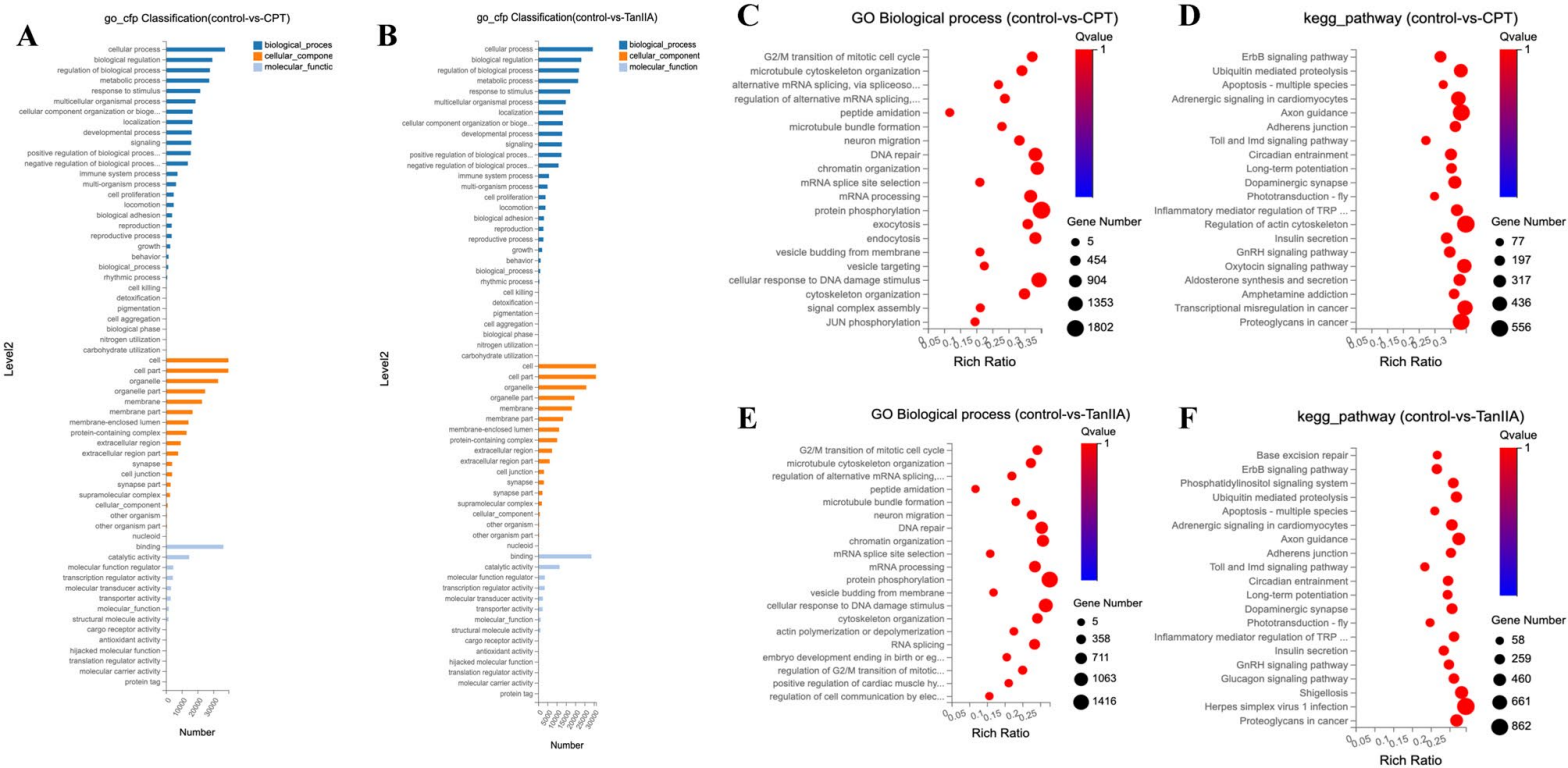
Functional annotation and pathway enrichment analysis of DEGs (**A**) GO enrichment of the commonly detected DEGs of the Control versus the CPT group, which included molecular function, cellular components, and biological processes. (**B**) Enrichment of the commonly detected DEGs of the Control versus the TanIIA group, which included molecular function, cellular components, and biological processes. (**C**) GO Biological process of DEGs of the Control versus the CPT group. (**D**) GO Biological process of DEGs of the Control versus the TanIIA group. (**E**) KEGG pathway enrichment bubble map of DEGs of the Control versus the CPT group, where a larger p-value (−log10) indicates a higher degree of enrichment. (**F**) KEGG pathway enrichment bubble map of DEGs of the Control versus the TanIIA group, where a larger p-value (−log10) indicates a higher degree of enrichment.

GO enrichment analysis showed that CPT and TanIIA mainly affected the biological processes of the cells (Fig. 6A and B). Biological processes analysis of using CPT before compared with after revealed DEGs in cytoskeleton organization, G2/M transition of mitotic cell cycle, protein phosphorylation, and others related to cell metabolism and migration. (Fig. 6C). KEGG enrichment analysis showed similar changes in Environmental Information Processing, Genetic Information Processing, and Organismal Systems after CPT reached cellular process. Also, we observed enrichment in Adherens junction, Ubiquitin mediated proteolysis, Toll and Imd signaling pathway, Proteoglycans in cancer, and others involved in cellular process (Fig. 6D). We next explored the biological processes that were strongly associated with A549 when treated with TanIIA. We found G2/M transition of mitotic cell cycle, protein phosphorylation, actin polymerization or depolymerization, cytoskeleton organization, and others related to cell metabolism, such as lipid metabolic process (Fig. 6E). For KEGG enrichment included Adherens junction, Apoptosis—multiple species, and others related to Signal transduction, Endocrine system, Development and regeneration, Cell growth and death, Cellular community—eukaryotes and so on (Fig. 6F).

### Effects of CPT and TanIIA on ZO1 and occludin protein and mRNA expression in A549 cells

**Figure 7:**
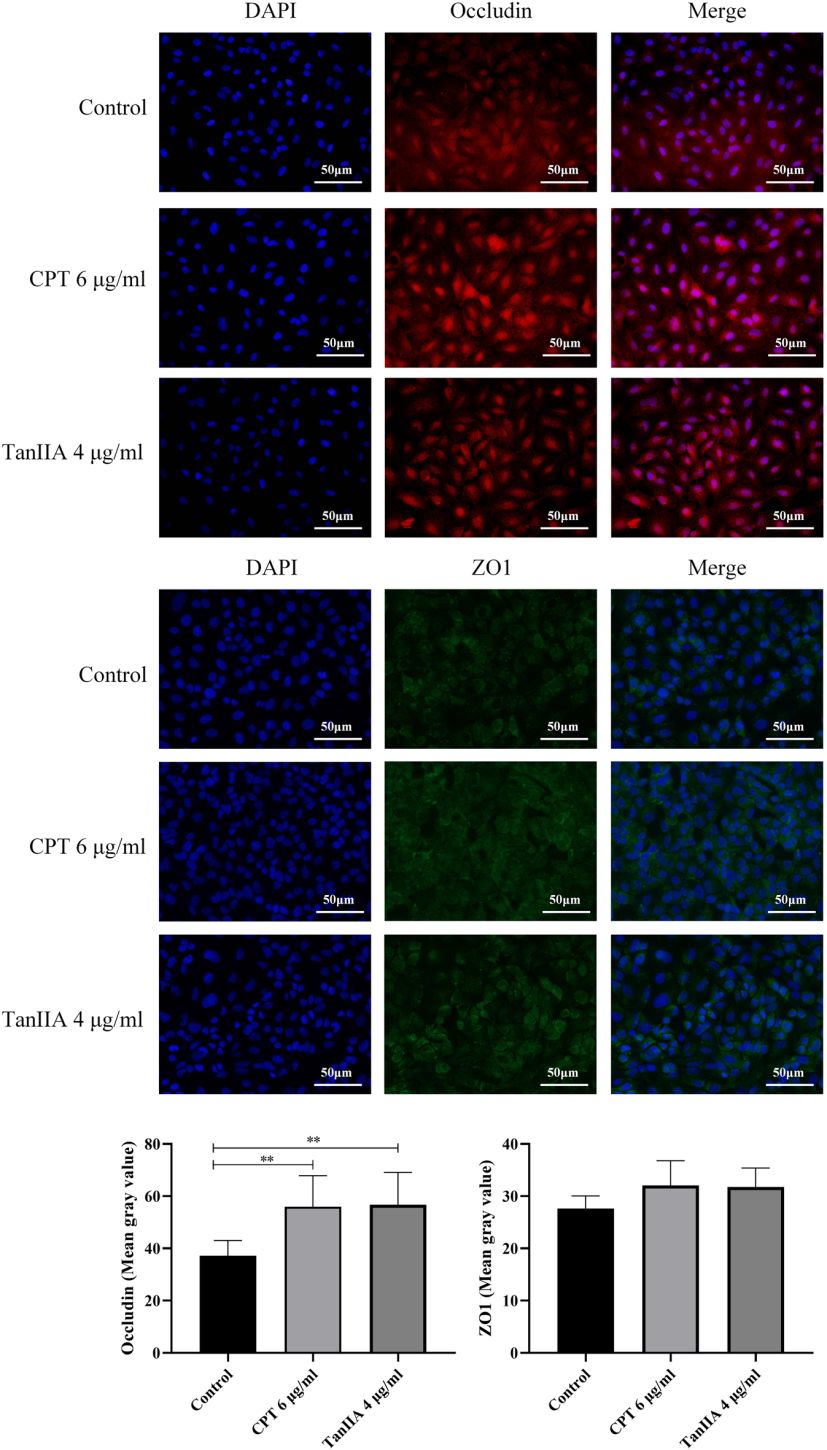
Effects of CPT and TanIIA on ZO1 and Occludin expression in A549 cells. Cell immunofluorescence assay following incubation of A549 cells with 6 μg/ml CPT and 4 μg/ml TanIIA for 24 h was performed to determine ZO1 and Occludin expression levels. Magnification ×400. Values are presented as the mean value ± standard deviations of the mean. **P < 0.01 compared with control group were recognized as statistically significant.

To further confirm the mechanism through which CPT and TanIIA functions, ZO1 and Occludin protein expression was detected with immunofluorescence assay. As shown in Fig. 7, Occludin protein expression in the CPT and TanIIA group was significantly induced compared to that in the control group (*P* < 0.01). The expression of the ZO1 proteins was not significantly different between the CPT or TanIIA treated group and control group. The WB experiment was carried out for the further demonstrate the effect of CPT and TanIIA on ZO1 and Occludin protein expression.

**Figure 8:**
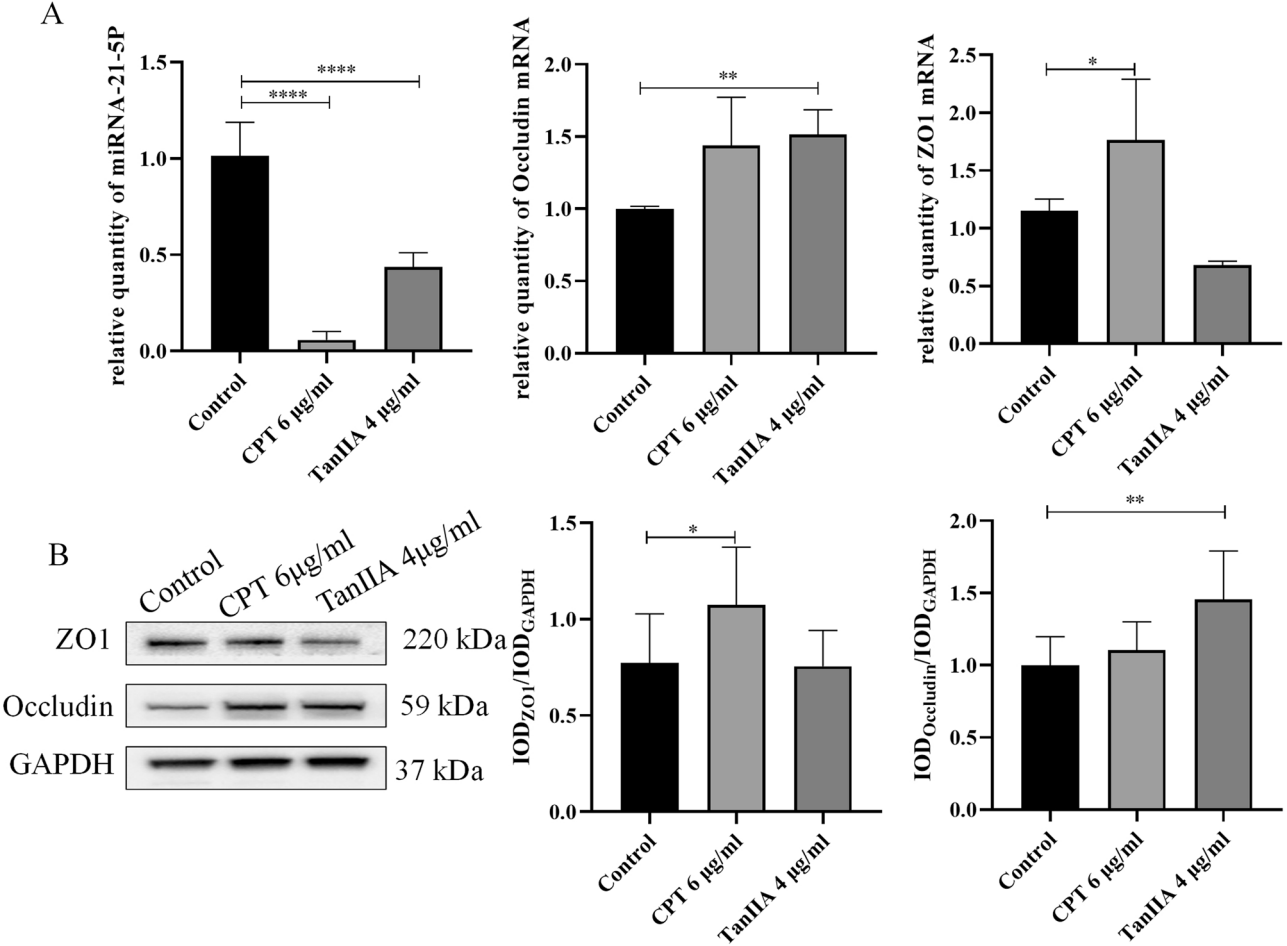
Effects of CPT and TanIIA on miR‐21 as well as ZO1 and Occludin expression in A549 cells. (**A**) Reverse transcription quantitative polymerase chain reaction using TaqMan^®^ miRNA assay following incubation of A549 cells with 6 μg/ml CPT and 4 μg/ml TanIIA for 24 h was performed in order to determine miR‐21-5p, ZO1 and Occludin expression levels. (**B**) Western blot analysis of ZO1 and Occludin protein expression levels in A549 cells following treatment with 6 μg/ml CPT and 4 μg/ml TanIIA for 24 h. GAPDH was used as the internal control. We completed the Western-blot experiment shown in this picture in the same gel. However, in order to incubate different antibodies, the PVDF membrane was therefore sheared after the transfer of one complete gel. The raw Western-blot imagines were demonstrated in the Supplement Fig. 8. We repeated the experiment three times on a single gelatin plate, while blotting images are provided for each of the three different exposure times, and white plate images are provided at the end. Values are presented as the mean value ± standard deviations of the mean. ****P < 0.0001, **P < 0.01, *P < 0.05 compared with control group were recognized as statistically significant.

Figure 8A showed that Occludin mRNA expression in the A549 cells were dramatically increased in TanIIA treatment compared to that in monocultures A549 cell (*P* < 0.01), while treatment with CPT significantly increased ZO1 levels (*P* < 0.05). As shown in Fig. 8B, the WB analysis showed that ZO1 was significantly raised in CPT treated cells, compared to that in monocultures cell (*P* < 0.05), whereas TanIIA treatment dramatically increased the Occludin in A549 cells (*P* < 0.01).

**Figure 9:**
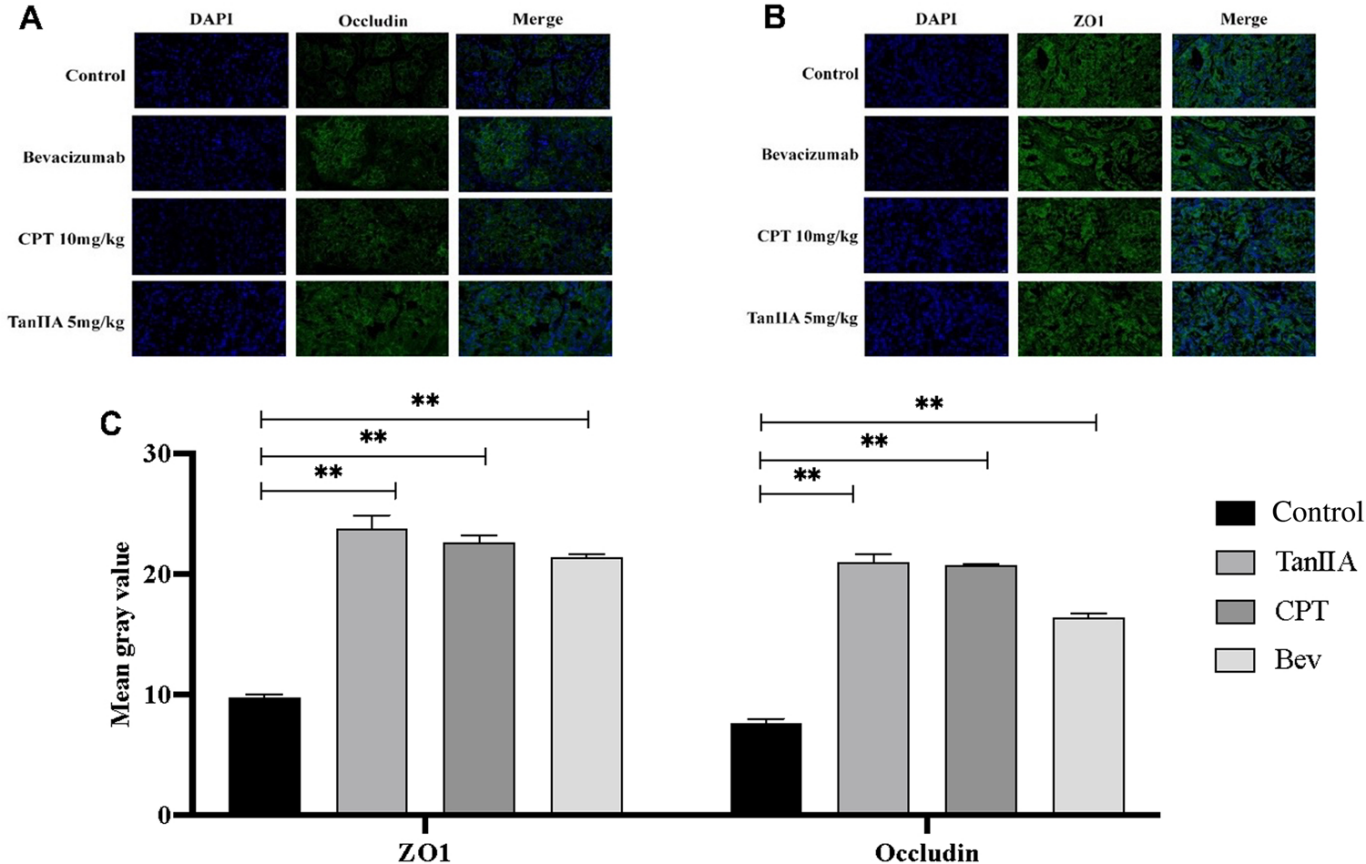
Expression of ZO1and Occludin protein in tumor tissues of various groups of mice. By immunofluorescence staining, it was found that the staining fluorescence intensity of Occludin protein and ZO1 protein of the tumor tissues in the administered group was stronger than that of the control group, indicating that the protein expression was increased. Compared with the control group, **P < 0.01.

By immunofluorescence staining, it was found that the staining fluorescence intensity of Occludin protein and ZO1 protein of the tumor tissues in the administered group was stronger than that of the control group, indicating that the protein expression was increased, as shown in Fig. 9.

**Figure 10:**
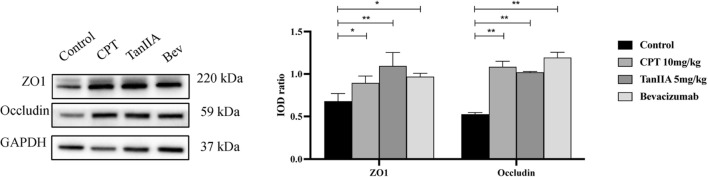
Expression of Occludin and ZO1 protein in tumor tissues of mice in each group. The results by WB showed that administration group of 10 mg/kg CPT and 5 mg/kg TanIIA could significantly increase the expression of Occludin and ZO1 protein in the tumor tissues of mice (*P* < 0.05 and *P* < 0.01). We completed the WB experiment shown in this picture in the same gel. However, in order to incubate different antibodies, the PVDF membrane was therefore sheared after the transfer of one complete gel. The raw Western-blot imagines were demonstrated in the Supplement Fig. 10. We repeated the experiment two times on a single gelatin plate, while blotting images are provided for each of the three different exposure times, and white plate images are provided at the end. Compared with the control  group, **P < 0.01, *P < 0.05.

The results by WB showed that administration group of 10 mg/kg CPT and 5 mg/kg TanIIA could significantly increase the expression of Occludin and ZO1 protein in the tumor tissues of mice (*P* < 0.05 and *P* < 0.01), see Fig. 10.

### Effect of CPT and TanIIA on Occludin protein and ZO1 protein in tumor tissues of A549 transplanted tumor-bearing mice

miR-21 is involved in the progression of a variety of cancers, and its expression is often up-regulated in cancers. Combined with our RNA-Seq results, the further studies were proceeded to clarify the effect of miR-21-5p on A549 cell differentiation and the regulatory function of CPT and TanIIA. As shown in Fig. 8A**,** miR-21-5p expression in CPT and TanIIA group was remarkable downregulated compared to the control group (*P* < 0.0001).

**Figure 11:**
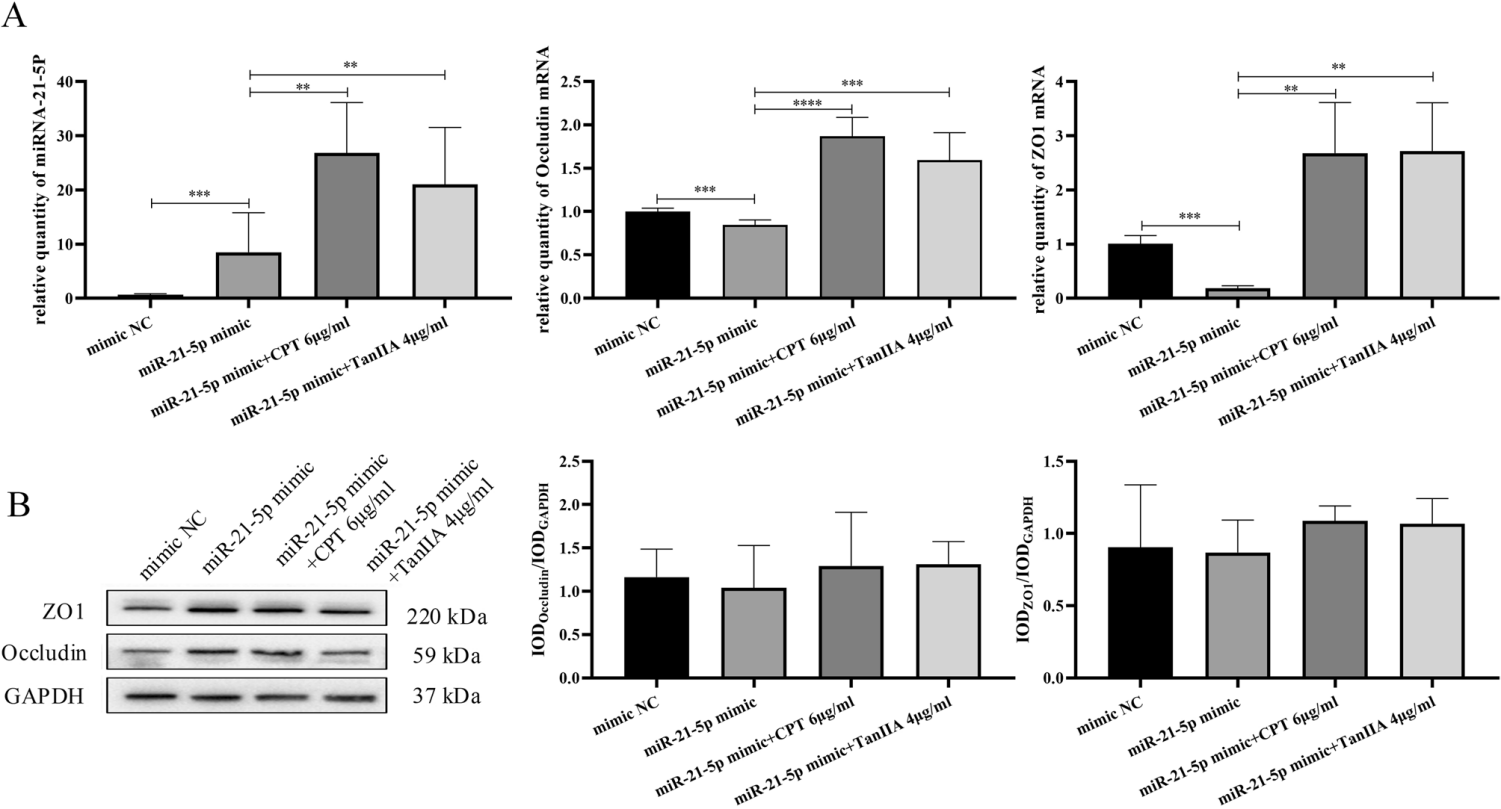
Effects of CPT and TanIIA on transient enforced expression of miR-21-5p mimics in A549 cell lines. (**A**) Reverse transcription quantitative polymerase chain reaction using TaqMan^®^ miRNA assay following incubation of A549 cells with 6 μg/ml CPT and 4 μg/ml TanIIA for 24 h was performed in order to determine miR‐21-5p, ZO1 and Occludin expression levels. (**B**) Western blot analysis of ZO1 and Occludin protein expression levels in A549 cells following treatment with 6 μg/ml CPT and 4 μg/ml TanIIA for 24 h. GAPDH was used as the internal control. We completed the Western-blot experiment shown in this picture in the same gel. However, in order to incubate different antibodies, the PVDF membrane was therefore sheared after the transfer of one complete gel. The raw Western-blot imagines were demonstrated in the Supplement Fig. 11. We repeated the experiment two times on a single gelatin plate, while blotting images are provided for each of the three different exposure times, and white plate images are provided at the end. Values are presented as the mean value ± standard deviations of the mean. ****P < 0.0001, ***P < 0.001, **P < 0.01 compared with control group were recognized as statistically significant.

Moreover, the transient enforced expression of miR-21-5p mimics was transfected into A549 cell lines. As shown in Fig. 11A**,** the level of miR-21-5p in the cells were dramatically increased in miR-21-5p mimics transfected group, compared to that mimic control group (*P* < 0.001), while the mRNA expressions of ZO1 and Occludin were significantly decreased in miR-21-5p mimics transfected group, compared to that mimic control group (*P* < 0.001). The expression of miR-21-5p, ZO1 and Occludin in the cells, that transfected in miR-21-5p mimics and treated with 6 μg/ml of CPT and 4 μg/ml of TanIIA, were all significantly increased, compared to monocultures miR-21-5p mimics transfected cells. The protein expressions of ZO1 and Occludin were increased in cells transfected miR-21-5p mimics and treated with 6 μg/ml of CPT and 4 μg/ml of TanIIA, but there were no significant changes in each groups (Fig. 11B).

## Discussions

Despite many advances in the treatment of NSCLC, the prognosis of patients is still poor. The main reason is that most patients are diagnosed with advanced lung cancer with local invasion and metastasis to regional lymph nodes or distant sites^28^. Therefore, in-depth investigation of the molecular mechanism of the occurrence and development of NSCLC, besides exploration of new therapeutic strategies, will greatly improve the prognosis of patients.

Danshen is a well-known TCM with a wide range of cardiovascular and cerebrovascular protective effects and has been used in Asian countries for many centuries. Some studies have reported that the active components of Danshen have anti-tumor pharmacological effects, among which TanIIA and CPT are representative^29,30^. TanIIA is a phenoquinone derivative extracted from *Salvia miltiorrhiza Bge*., which is often used in the treatment of cardiovascular diseases^31^. However, cumulative evidence from preclinical and clinical studies confirms that TanIIA has favorable antitumor properties^32^. Several studies have shown that it can inhibit the growth of various tumor cell lines^33–35^. TanIIA showed certain cytotoxic effects both in vivo and in vitro^25,36^. In addition, CPT is a diterpenoid monomer, which is a lipid soluble component extracted from Danshen. CPT has been shown to have antioxidant, antitumor, antibacterial and anti-inflammatory biological activities. In recent years, many researchers have paid attention to its anti-tumor pharmacological activity^37^. CPT can exert its anti-tumor activity by inhibiting tumor cell migration and invasion, inducing tumor cell apoptosis, inhibiting the generation of new blood vessels, inhibiting tumor cell proliferation, regulating androgen receptor signaling, inhibiting lymphangiogenesis and so on^38^. In this study, CPT and TanIIA were used to intervene A549 cells, and it was found that the inhibition rates of both on A549 were time-dependent and dose-dependent. At the same time, the 48 h IC50 value was calculated for subsequent studies. After the intervention of CPT and TanIIA, the migration ability of A549 cells decreased, and the level of apoptosis increased.

In order to explore the mechanism of CPT and TanIIA inhibiting A549 cells, we performed RNA-Seq on the cells after intervention. Comparison of differential gene expression levels of small RNA showed that both CPT and TanIIA could lead to differential gene expression including miR-21-5p. For the results of GO and KEGG analysis after CPT and TanIIA intervention in A549 cells, in addition to a wide range of pathways, we also found several pathways involved in protein phosphorylation, transmembrane transport, cell migration, Cell differentiation and G2/M transition of mitotic cell cycle, e.g. Adherens junction, Apoptosis—multiple species, Toll and Imd signaling pathway, ErbB signaling pathway and so on. The results combined with RNA-Seq suggest that further studies should be conducted to clarify the effect of miR-21-5p on cell differentiation and the regulatory role of CPT and TanIIA.

Previous studies have shown that miR-21 is involved in the progression of many cancers, such as gastric cancer, colon cancer, renal cell carcinoma, esophageal cancer, etc., and its expression is up-regulated in many cancers^39–41^. Ren et al. suggested that miR-21-5p may promote cell proliferation, inhibit cell apoptosis and promote the development of lung cancer in lung cancer A549 cells^42^. To further explore the phenotypic characteristics of miR-21-5p up-regulation in promoting tumor proliferation and migration, we systematically studied the relevant literature. In previous studies on the regulation of intestinal epithelial barrier function by miR-21-5p level, it was found that miR-21-5p could promote the increase of intestinal epithelial permeability by down-regulating the expression level of tight junction protein (TJ)^43,44^. Tight junctions refer to the close association of cell membranes between cells to form an impermeable barrier^45^. Transmembrane TJ (Occludin, Claudins, etc.) form paracellular seals through interactions between extracellular domains and cytoplasmic plaques through interactions between intercellular domains and various adaptor proteins (such as ZO-1)^46^. As the first transmembrane tight junction protein identified, Occludin is essential for TJ function. Numerous studies have shown that Occludin plays an important role in maintaining cellular barrier function, as well as in promoting skin wound healing^47^, maintaining proximal tubular epithelial function^48^, neovascularization and angiogenesis^49^. Tobioka et al.^50^ found that Occludin protein was positive in normal lung tissues and lung adenocarcinoma tissues, while it was negative in squamous cell carcinoma, large cell carcinoma, small cell carcinoma or large cell neuroendocrine carcinoma. Abnormal expression of Occludin protein may be involved in the occurrence of NSCLC. The main characteristics of tumor cells are continuous proliferation and high metastatic potential^51^. Wang et al.^52^ demonstrated that Occludin knockdown promotes apoptosis and reduces invasion ability of lung cancer cells.

In our study, both CPT and TanIIA could significantly reduce the expression level of miR-21-5p after intervention in normal A549 cells. In addition, TanIIA significantly increased the expression levels of Occludin mRNA and protein in A549 cells, and the same trend was also observed in CPT group. CPT could increase the expression levels of ZO1 mRNA and protein in A549 cells, while TanIIA group showed a downward trend. To further explore the correlation between miR-21-5p expression and cell TJ (Occludin), as well as cohesion related proteins (ZO1), miR-21-5p was overexpressed in A549 cells by cell transfection technology, and it was found that the mRNA levels of Occludin and ZO1 decreased accordingly. Cell migration and proliferation were also enhanced. After drug intervention, CPT or TanIIA could significantly increase the mRNA levels of Occludin and ZO1 in miR-21-5p overexpressing A549 cells. Meanwhile, the protein expression levels of Occludin and ZO1 also showed an increasing trend.

## Conclusion

In conclusion, both CPT and TanIIA significantly inhibited the proliferation of A549 tumour cells and tumour tissue growth in animal models. The preliminary findings may be related to the targeting of miR-21-5p, which plays a role in increasing the expression levels of cohesion-associated proteins (Occludin and ZO1), and in particular the transcription and expression levels of the Occludin protein, which provides a new strategy for expanding the scope of tumour therapy. However, our study still has some limitations: the TanIIA group did not significantly exert a regulatory effect on the expression level of ZO1, but whether it has an effect on the upstream genes of ZO1 protein needs to be further clarified. In this study, we explored and obtained Chinese herbal components widely used in the treatment of cardiovascular diseases, which also have anti-tumour effects, and further explored their mechanisms of action, which can help to provide new strategies for the prevention and treatment of lung cancer.

### Supplementary Information

Supplementary Figures.

Supplementary Legends.

Supplementary Table S1.

Supplementary Table S2.

Supplementary Table S3.

Supplementary Table S4.

Supplementary Table S5.

Supplementary Table S6.

Supplementary Table S7.

## Data Availability

The authors confirm that the data supporting the findings of this study are available within the article.

## Abbreviations

ANOVA: One-way analysis of variance
BP: Biological processes
CC: Cellular component
CPT: Cryptotanshinone
DEGs: Differentially expressed genes
ECL: Electrochemiluminescence
FCM: Flow cytometry
GO: Gene ontology
KEGG: Kyoto encyclopedia of genes and genomes
MF: Molecular function
miRNAs/miRs: MicroRNAs
NSCLC: Non-small cell lung cancer
SD: Standard deviation
TanIIA: Tanshinone IIA
TCM: Traditional Chinese medicine
