# Supplementary material for: Drug monomers from Salvia miltiorrhiza Bge. promoting tight junction protein expression for therapeutic effects on lung cancer
Source: Sci Rep. 2023 Dec 21;13:22928. doi: 10.1038/s41598-023-50163-8 (PMC10739844; doi:10.1038/s41598-023-50163-8)
Supplement: Supplementary file 1 — Supplementary Figures. [file 41598_2023_50163_MOESM1_ESM.pdf]

Figure 8

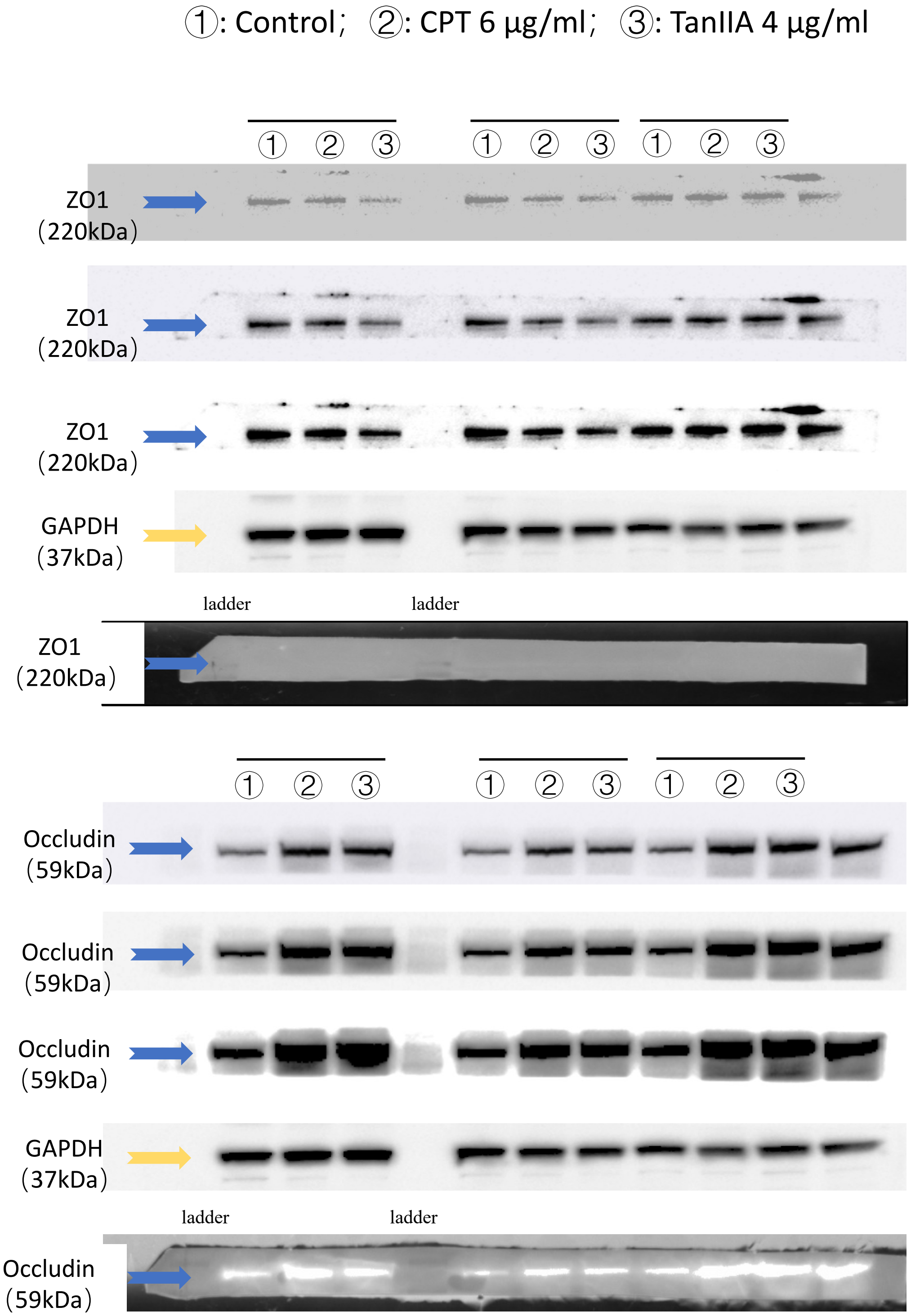

Figure legend:

Supplement Figure 8. We completed the Western-blot experiment shown in this picture in the same gel. However, in order to incubate different antibodies, the PVDF membrane was therefore sheared after the transfer of one complete gel. The raw Western-blot analyses of tight junction proteinsexpression, including ZO1 and Occludin, in A549 cells from control, CPT, and TanIIA groups were demonstrated. We repeated the experiment three times on a single gelatin plate, while blotting images are provided for each of the three different exposure times, and white plate images are provided at the end.

Figure 10

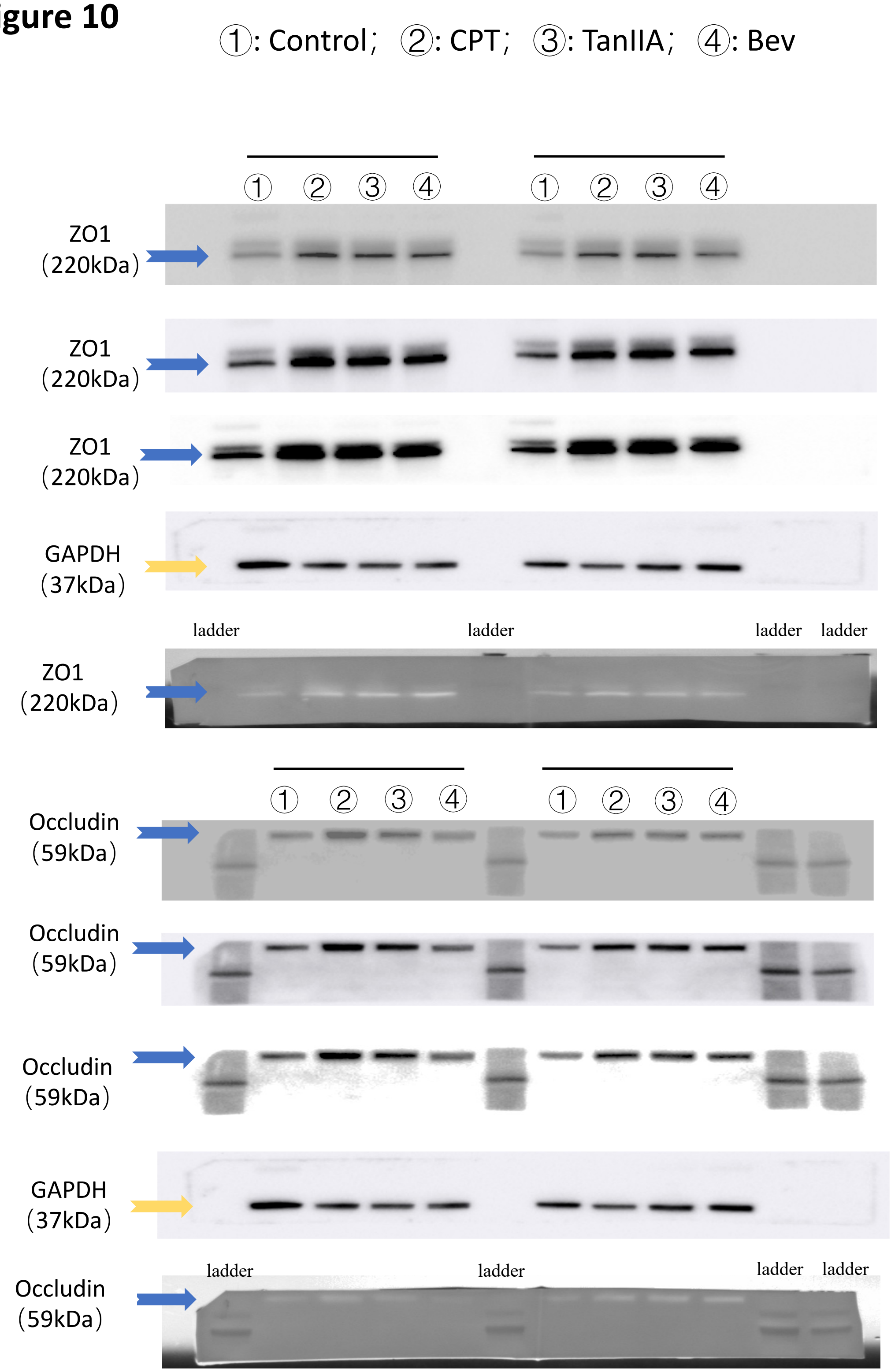

Figure legend:
Supplement Figure 10. We completed the Western-blot experiment shown in this picture in the same gel. However, in order to incubate different antibodies, the PVDF membrane was therefore sheared after the transfer of one complete gel. The raw Western-blot analyses of tight junction proteinsexpression, including ZO1 and Occludin, in tumor tissues of mice from control, CPT, TanIIA and bevacizumad groups were demonstrated . We repeated the experiment two times on a single gelatin plate, while blotting images are provided for each of the three different exposure times, and white plate images are provided at the end.

Figure 11

①: mimic NC; ②: miR-21-5p mimic;
③: miR-21-5p mimic+CPT 6 μg/ml ;
④: miR-21-5p mimic+ TanIIA 4 μg/ml

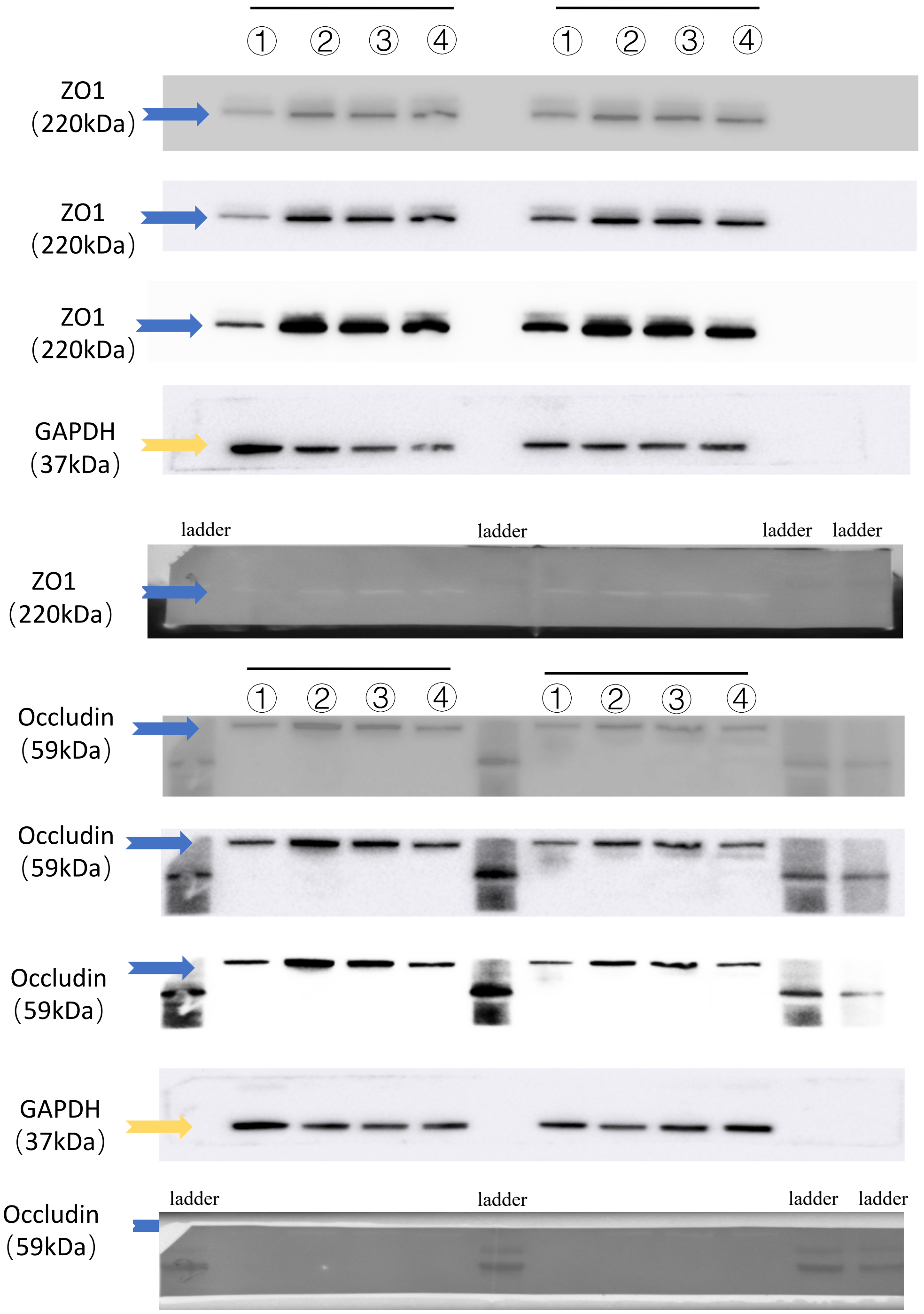

Figure legend:

Supplement Figure 11. . We completed the Western-blot experiment shown in this picture in the same gel. However, in order to incubate different antibodies, the PVDF membrane was therefore sheared after the transfer of one complete gel. The raw Western-blot analyses of tight junction proteinsexpression, including ZO1 and Occludin, of miR-21-5p mimics in A549 cell lines were demonstrated . We repeated the experiment two times on a single gelatin plate, while blotting images are provided for each of the three different exposure times, and white plate images are provided at the end.
