# Supplementary material for: Drug monomers from Salvia miltiorrhiza Bge. promoting tight junction protein expression for therapeutic effects on lung cancer
Source: Sci Rep. 2023 Dec 21;13:22928. doi: 10.1038/s41598-023-50163-8 (PMC10739844; doi:10.1038/s41598-023-50163-8)
Supplement: Supplementary file 2 — Supplementary Legends. [file 41598_2023_50163_MOESM2_ESM.docx]

**Table S1 Differential genes in the CPT and control groups.** There were 187 differentially expressed genes between the control and CPT groups.

**Table S2 Differential genes in the TanIIA and control groups.** There were 75 differentially expressed genes between the control and TanIIA groups.

**Table S3 Identical differential genes for CPT and TanIIA groups.** There were that CPT and TanIIA could act on 51 DEGs simultaneously showing in Venn diagrams.

**Table S4 Raw data for Figure 6A and 6C.** GO enrichment of the commonly detected DEGs of the Control versus the CPT group , which included molecular function, cellular components, and biological processes (6A). GO Biological process of DEGs of the Control versus the CPT group (6C).

**Table S5 Raw data for Figure 6B and 6E.** GO enrichment of the commonly detected DEGs of the Control versus the TanIIA group , which included molecular function, cellular components, and biological processes (6B). GO Biological process of DEGs of the Control versus the TanIIA group (6E).

**Table S6 Raw data for Figure 6D.** GO Biological process of DEGs of the Control versus the TanIIA group (6D).

**Table S7 Raw data for Figure 6F.** KEGG pathway enrichment bubble map of DEGs of the Control versus the TanIIA group, where a larger p-value (-log10) indicates a higher degree of enrichment (6F).
